# Supplementary material for: The dopamine receptor D5 gene shows signs of independent erosion in toothed and baleen whales
Source: PeerJ. 2019 Oct 11;7:e7758. doi: 10.7717/peerj.7758 (PMC6791347; doi:10.7717/peerj.7758)
Supplement: Supplemental Information 1 — Blue squares indicate the presence of DRD5 in genome annotation. Red squares indicate the absence of DRD5 annotation in the corresponding genome. Orthologous genes are joined by lines, each line representing a reference Bos taurus (cattle) DRD5 neighboring gene. Hippopotamus amphibius (hippopotamus) genes found in the same genomic scaffold are represented by dark gray squares and underlined with a bar indicating the corresponding accession number. Lipotes vexillifer (Yangtze River dolphin) syntenic map is incomplete due to the limited size of the corresponding explored genomic scaffold. [file peerj-07-7758-s001.docx]

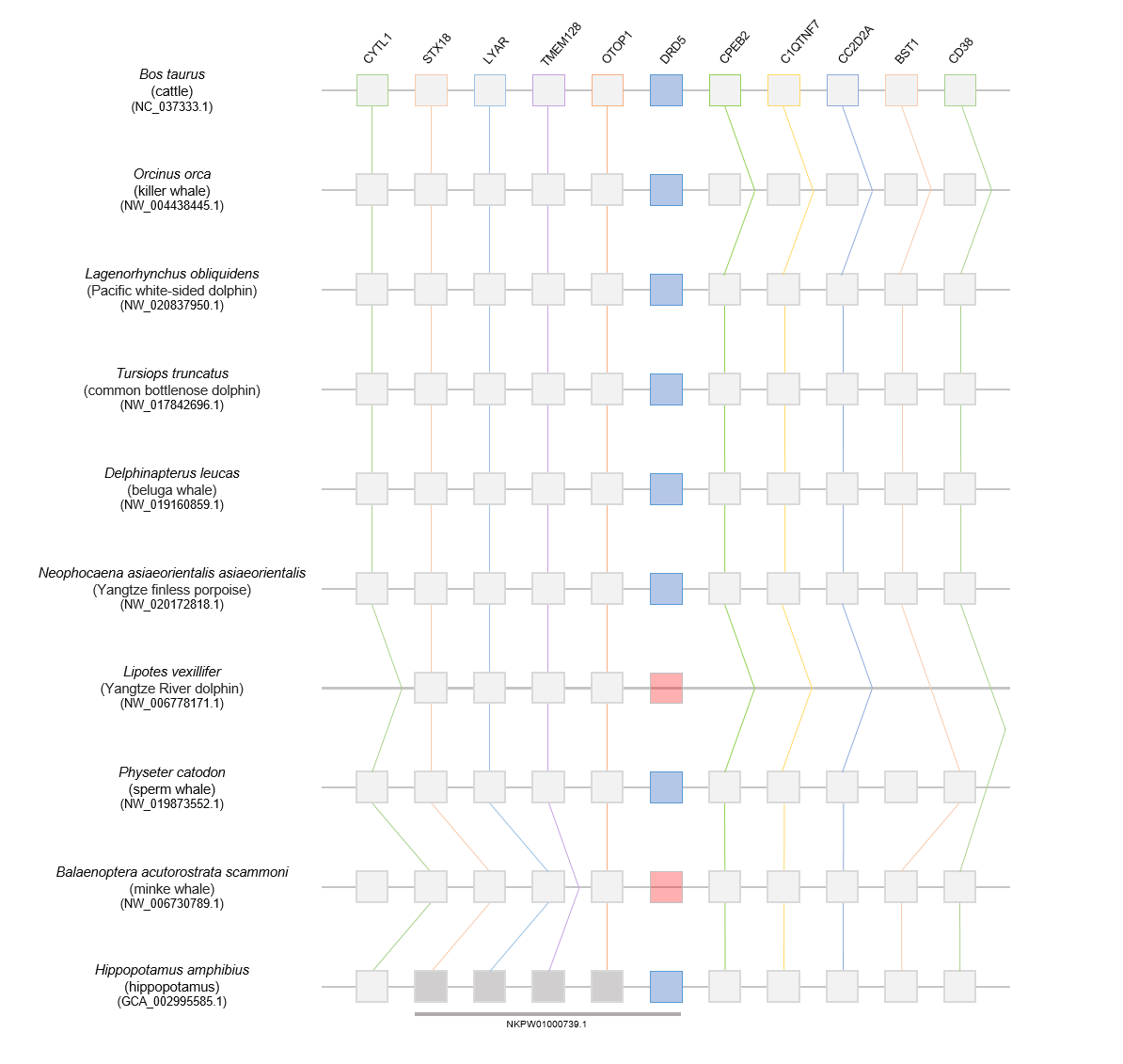
**Supplementary Figure 1: Comparative synteny maps of DRD_5_ genomic *locus* in Cetacea and *Hippopotamus amphibius* (hippopotamus).**

Blue squares indicate the presence of DRD_5_ in genome annotation. Red squares indicate the absence of DRD_5_ annotation in the corresponding genome. Orthologous genes are joined by lines, each line representing a reference *Bos taurus* (cattle) DRD_5_ neighboring gene. *Hippopotamus amphibius* (hippopotamus) genes found in the same genomic scaffold are represented by dark grey squares and underlined with a bar indicating the corresponding accession number. *Lipotes vexillifer* (Yangtze River dolphin) syntenic map is incomplete due to the limited size of the corresponding explored genomic scaffold.
