# Supplementary material for: The dopamine receptor D5 gene shows signs of independent erosion in toothed and baleen whales
Source: PeerJ. 2019 Oct 11;7:e7758. doi: 10.7717/peerj.7758 (PMC6791347; doi:10.7717/peerj.7758)

**Supplementary Figure 2: Multiple translation alignment of the NCBI annotated non ‘low-quality protein’ (LQ) tagged DRD_5_ coding sequences, including the manually predicted DRD_5_ coding sequence of *Hippopotamus amphibius* (hippopotamus) and excluding the NCBI DRD_5_ annotated coding sequence of *Tursiops truncatus* (common bottlenose dolphin).**

An alignment identity graph is presented above the alignment and *Homo sapiens* (human) DRD_5_ coding sequence is set as reference. Greenish colors represent very high alignment identity values, followed by yellowish (mid to high alignment identity values) and finally reddish colors, indicating a very low alignment identity value. Results show an average pairwise alignment identity of 83.6%.


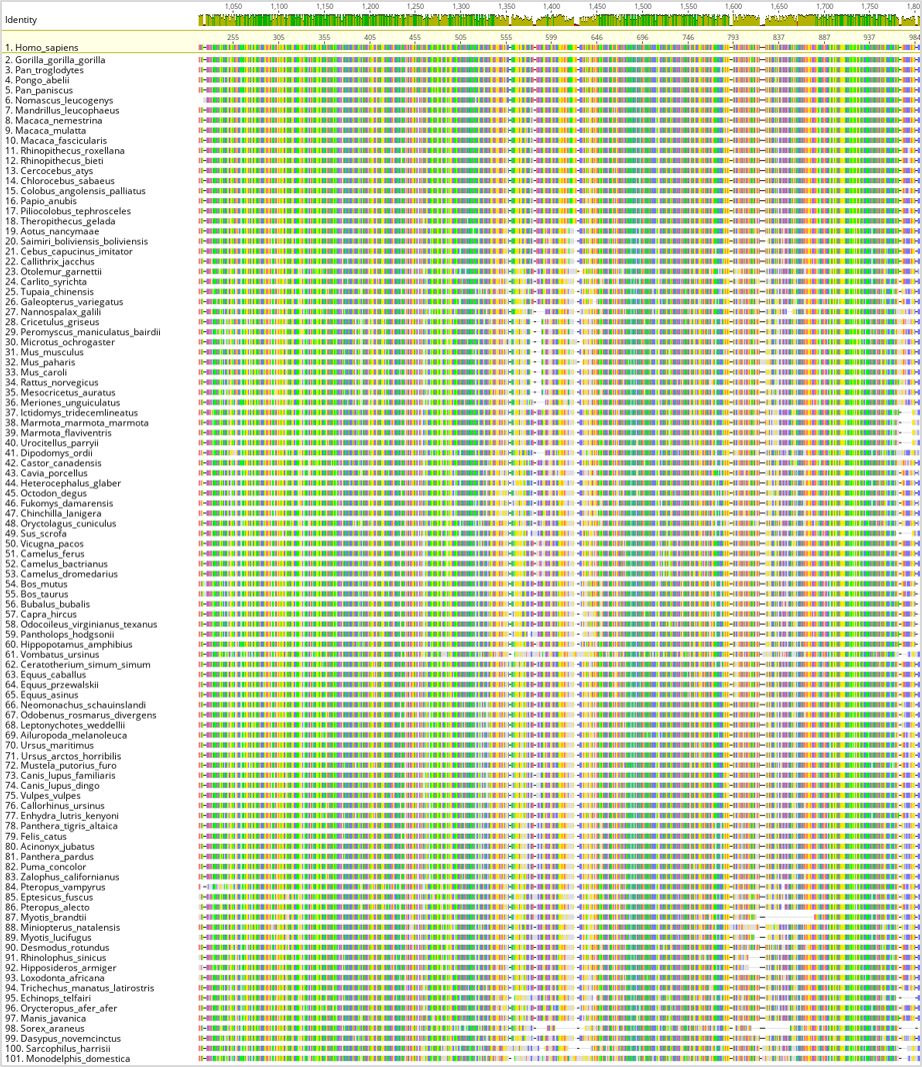

Supplement: Supplemental Information 2 — An alignment identity graph is presented above the alignment and Homo sapiens (human) DRD5 coding sequence is set as reference. Greenish colors represent very high alignment identity values, followed by yellowish (mid to high alignment identity values) and finally reddish colors, indicating a very low alignment identity value. Results show an average pairwise alignment identity of 83.6%. [file peerj-07-7758-s002.docx]
