# Supplementary material for: The dopamine receptor D5 gene shows signs of independent erosion in toothed and baleen whales
Source: PeerJ. 2019 Oct 11;7:e7758. doi: 10.7717/peerj.7758 (PMC6791347; doi:10.7717/peerj.7758)

Supplementary Material 1: SRA Validation of DRD_5 mutations in Cetacea.

Orcinus orca SRA confirmation of 2-nucleotide deletion

SRA experiments searched:

SRX188933 - PRJNA167475 - Baylor College of Medicine (BCM)

SRX466994 - PRJNA236163 - University of Durham

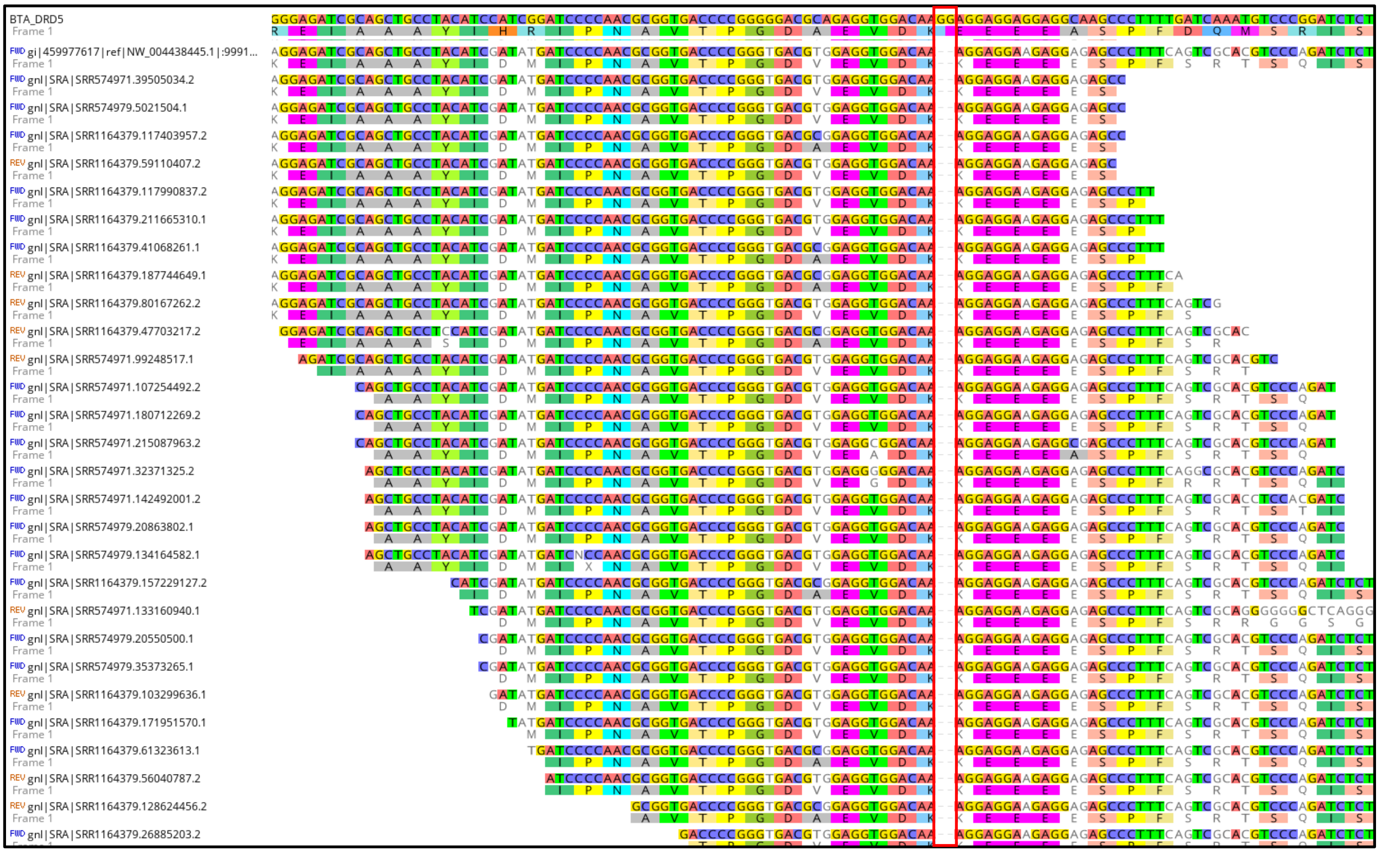

Lagenorhynchus obliquidens SRA confirmation of 2-nucleotide deletion

SRA experiments searched:

SRX4222897 - PRJNA475306 - BC Cancer Agency Michael Smith Genome Sciences Centre (BCCAGSC)

SRX4218967 - PRJNA475306 - BC Cancer Agency Michael Smith Genome Sciences Centre (BCCAGSC)

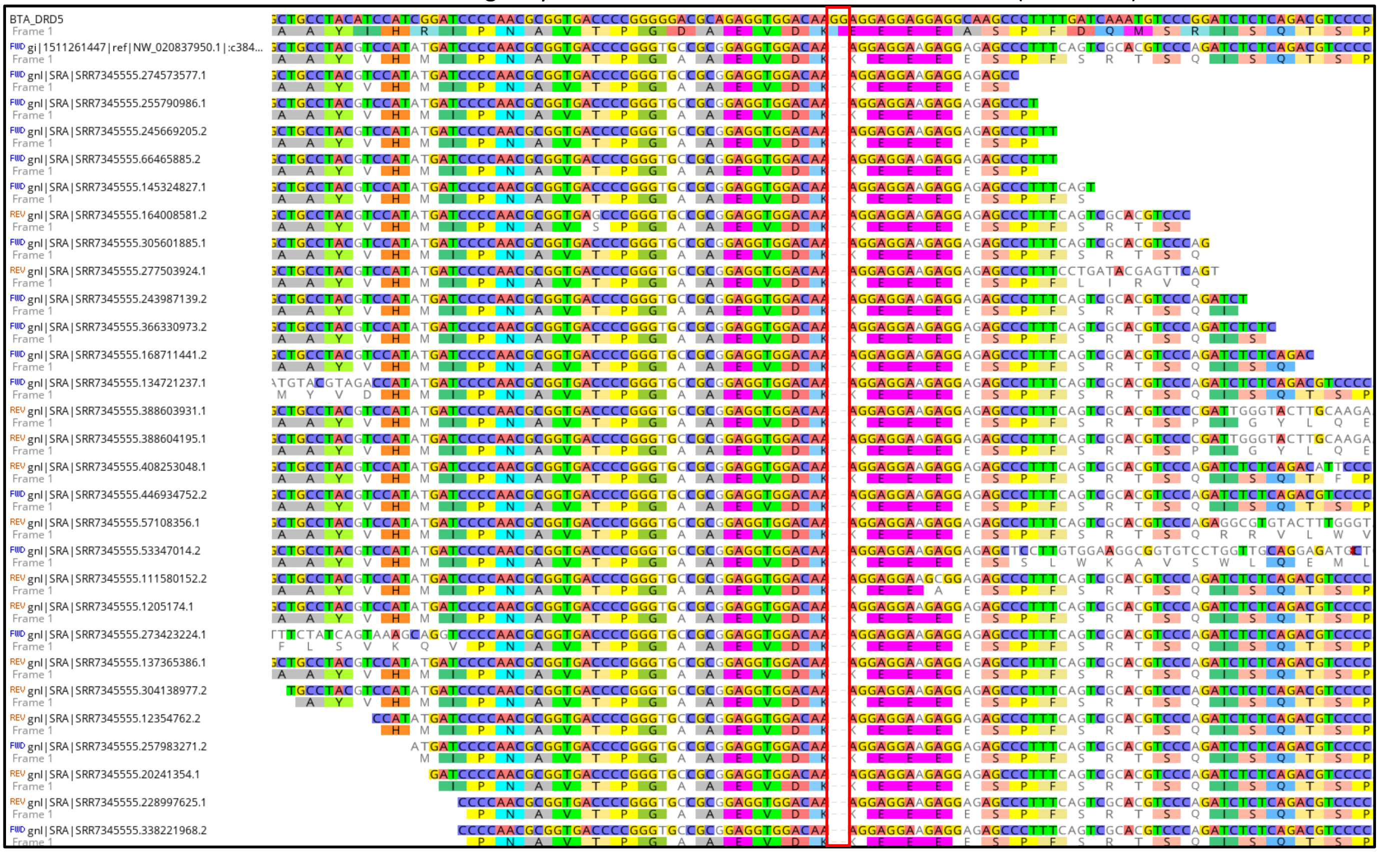

Tursiops truncatus SRA confirmation of 2-nucleotide deletion

SRA experiments searched:

SRX5097660 - PRJNA476133 - Johns Hopkins University

SRX200685 - PRJNA20367 - Baylor College of Medicine (BCM)

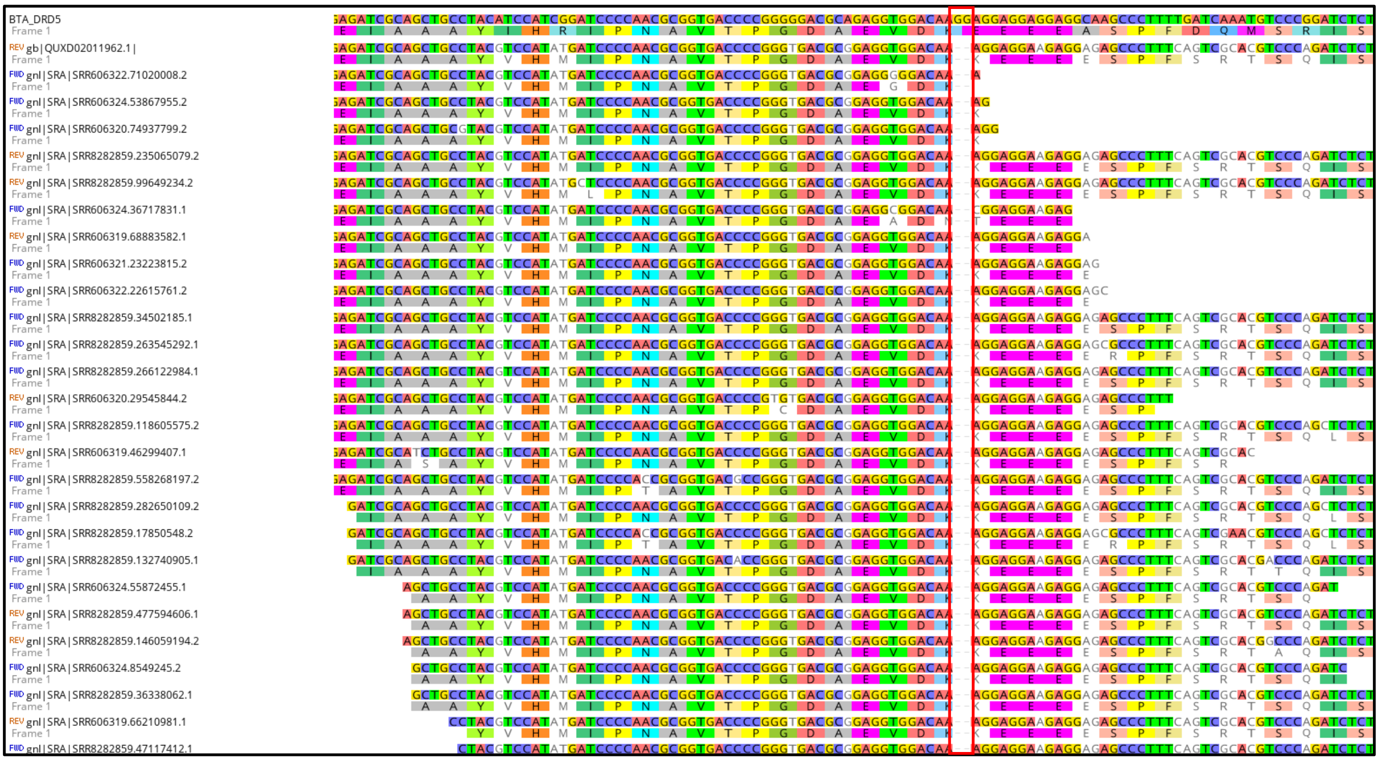

Delphinapterus leucas SRA confirmation of 1-nucleotide deletion

SRA experiments searched:

SRX2513439 - PRJNA360851 - BC Cancer Agency Michael Smith Genome Sciences Centre (BCCAGSC)

SRX2896241 - PRJNA360851 - BC Cancer Agency Michael Smith Genome Sciences Centre (BCCAGSC)

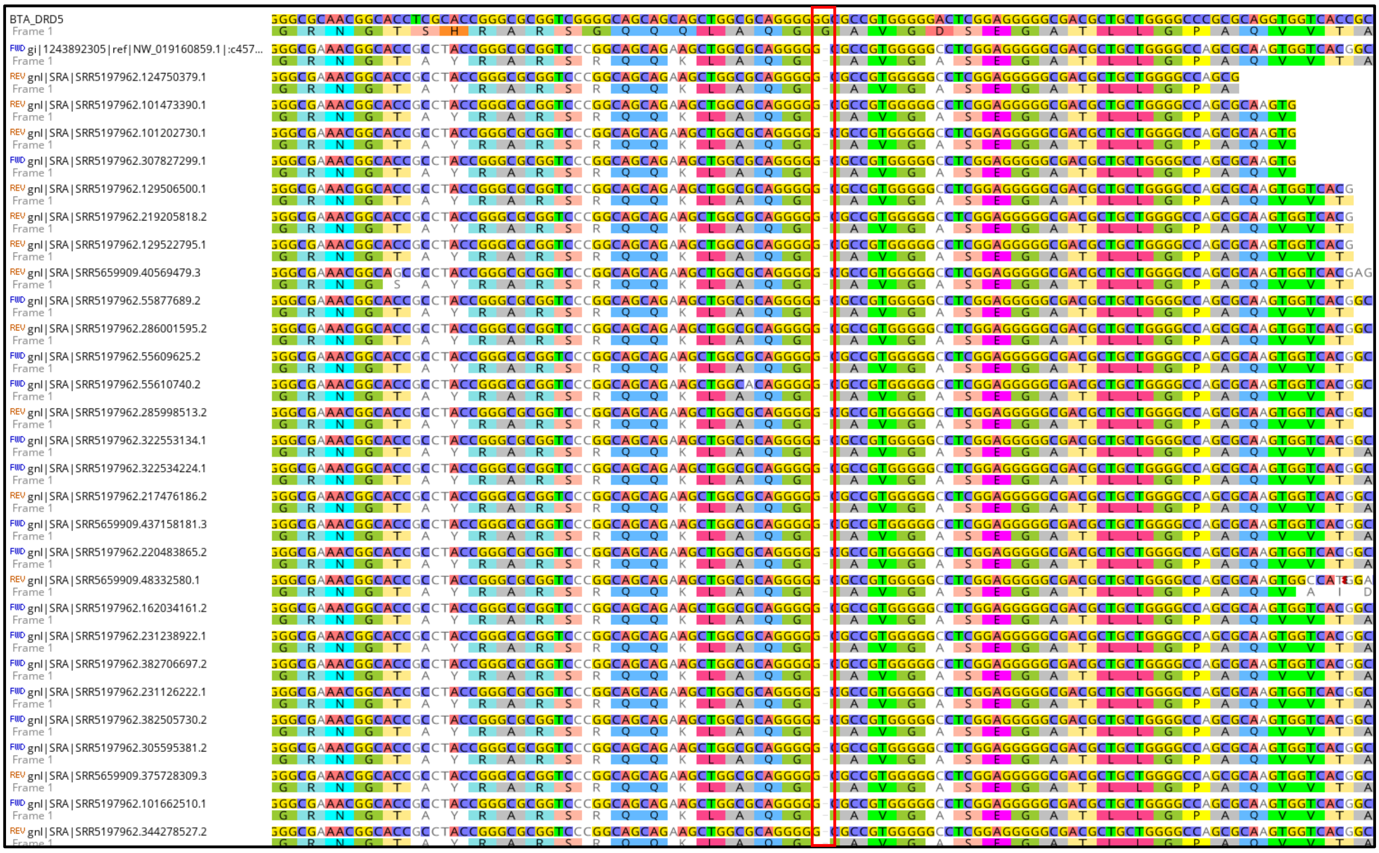

Neophocaena asieorientalis asiaeorientalis SRA confirmation of 2-nucleotide deletion

SRA experiments searched:

SRX3870625 - PRJNA433603 - Northwestern Polytechnical University

SRX2187761 - Beijing Genome Institute (BGI)

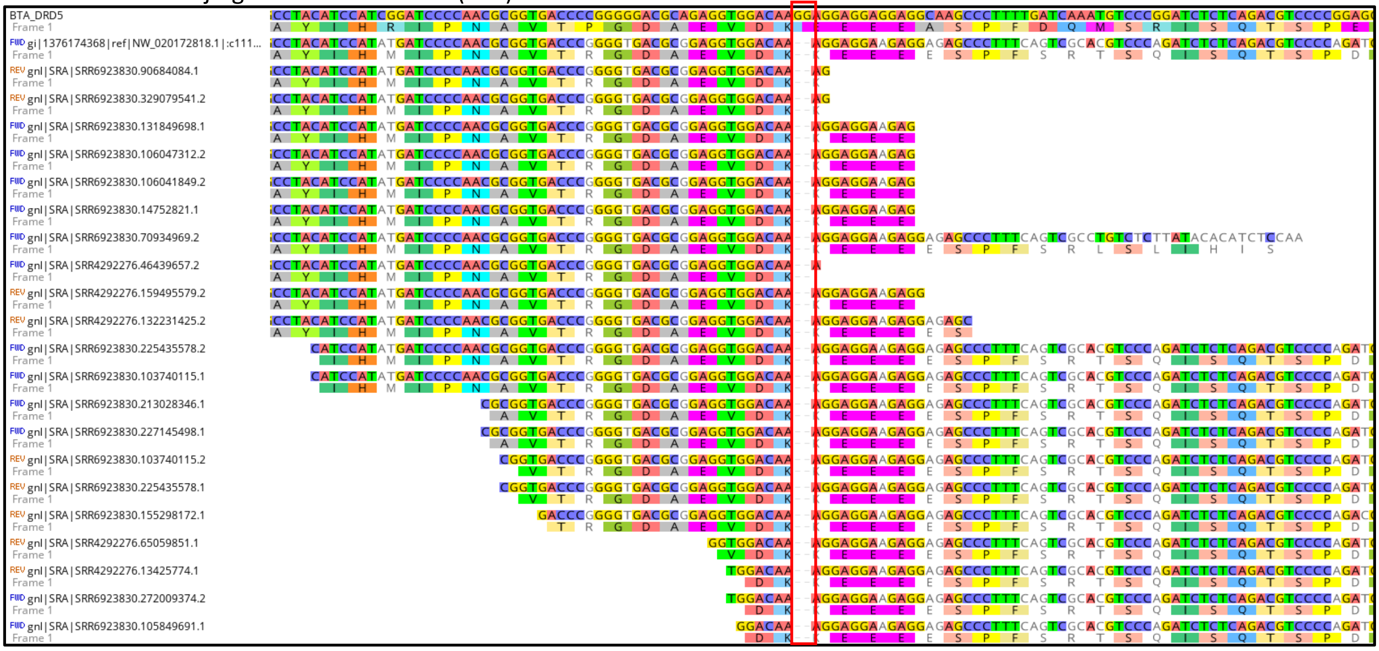

Physeter macrocephalus SRA confirmation of premature stop codon

SRA experiments searched:

SRX3230023 - PRJNA411766 - Beijing Genome Institute (BGI)

ERX2601910 - PRJEB24146 - CNGB

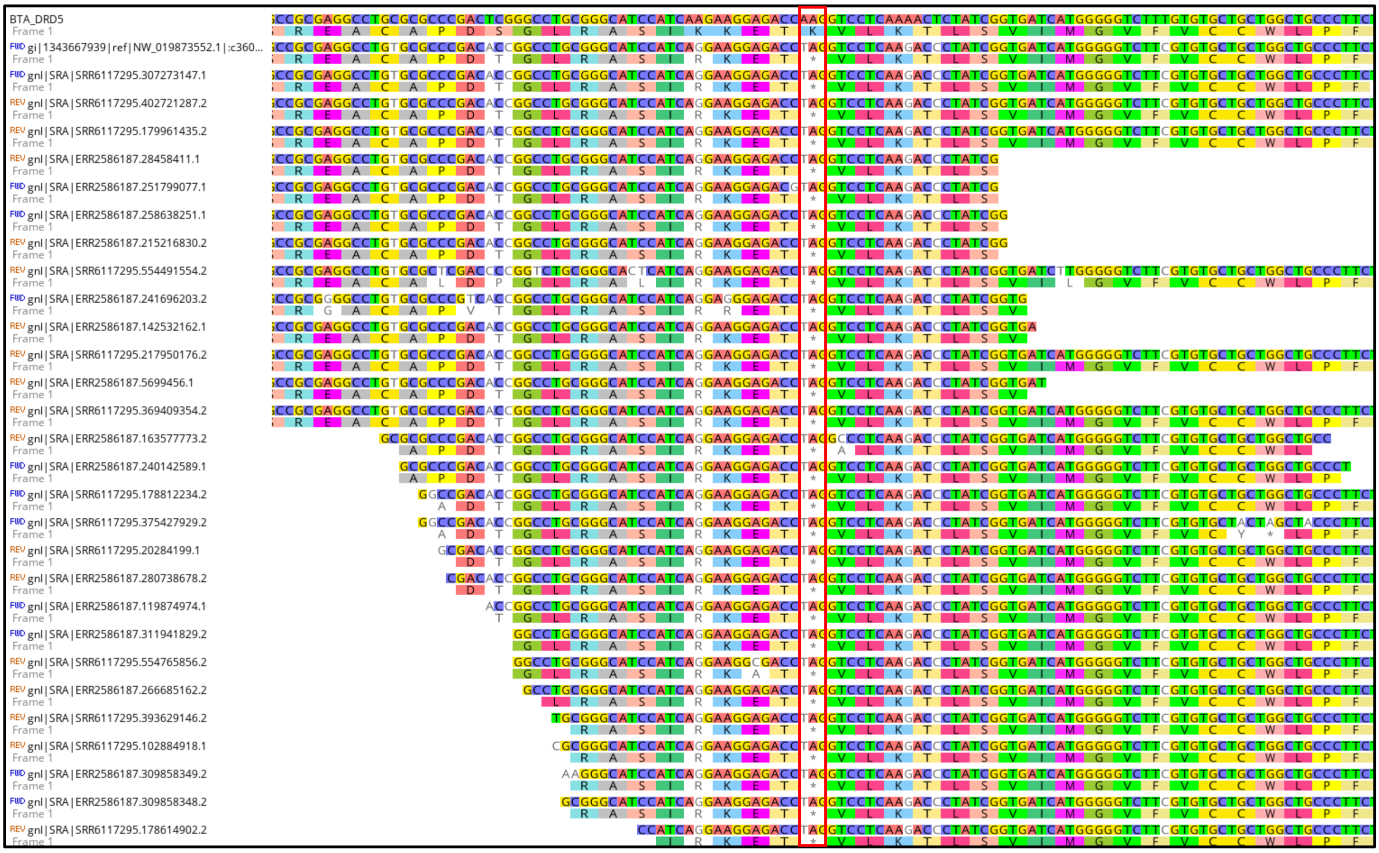

Balaenoptera bonaerensis SRA confirmation of 1-nucleotide deletion

SRA experiments searched:

SRX2007374 - PRJNA335553 - Institute of Marine Research

SRX2007375 - PRJNA335553 - Institute of Marine Research

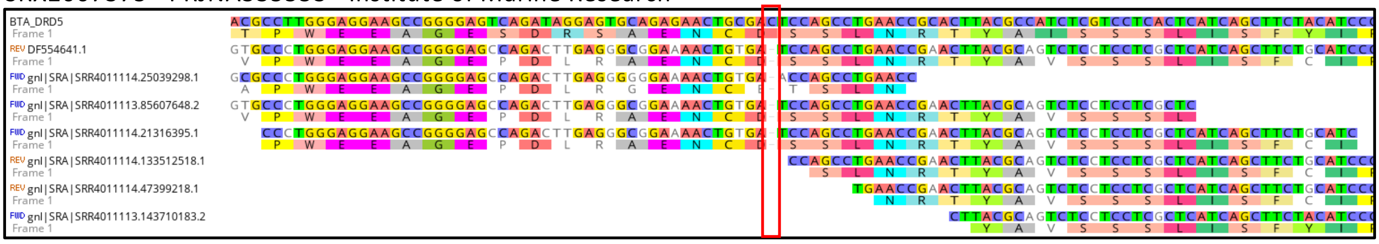

Eschrichtius robustus SRA confirmation of 1-nucleotide deletion

SRA experiments searched:

SRX2901264 - PRJNA389516 - Senckenberg Gesellschaft fuer Naturforschung

SRX2776065 - PRJNA384396 - Purdue University

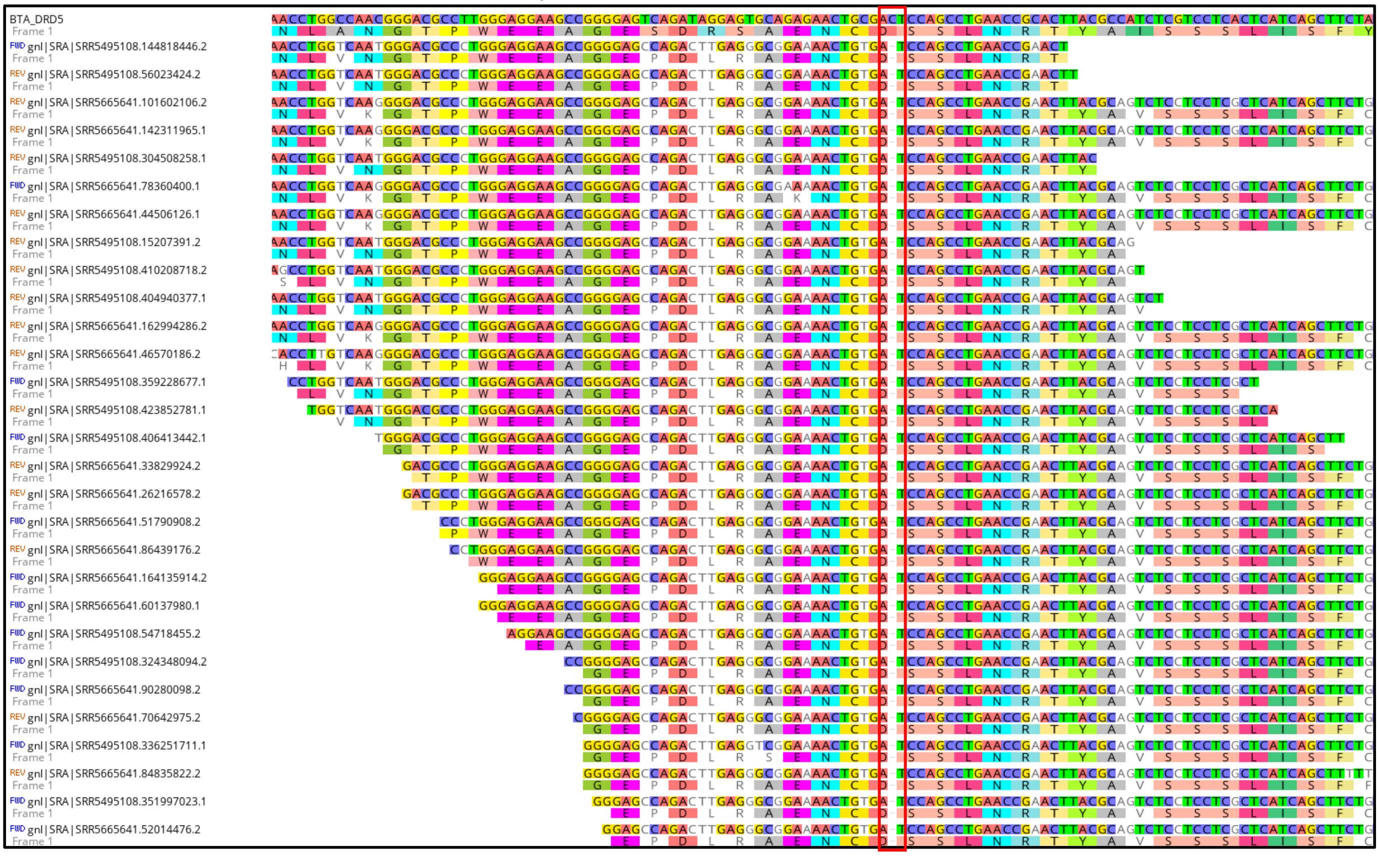

Balaena mysticetus SRA confirmation of 1-nucleotide deletion

SRA experiments searched:

SRX790318 - PRJNA194091 - University of Liverpool

SRX790317 - PRJNA194091 - University of Liverpool

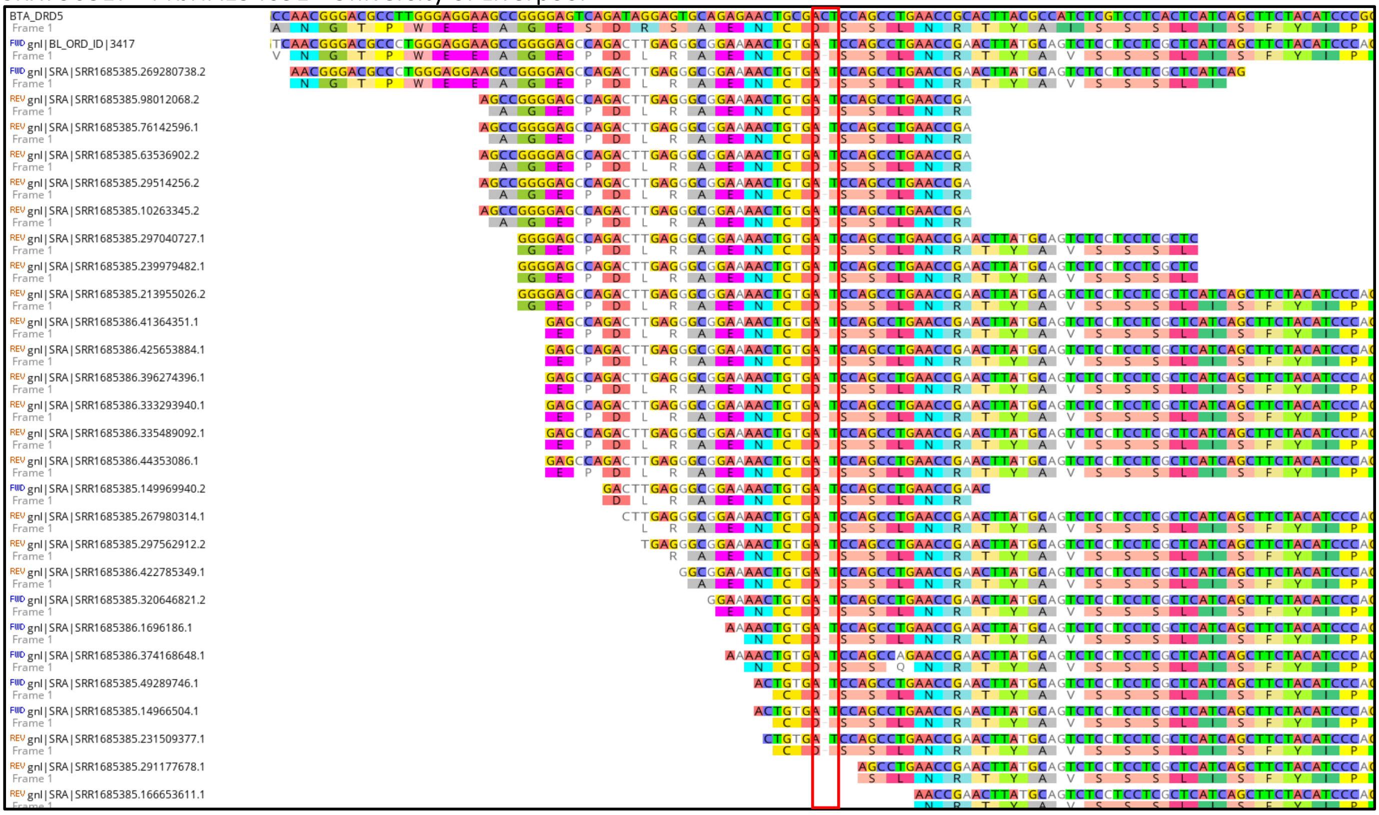

Supplement: Supplemental Information 3 — - [file peerj-07-7758-s003.pdf]
