# Supplementary material for: The dopamine receptor D5 gene shows signs of independent erosion in toothed and baleen whales
Source: PeerJ. 2019 Oct 11;7:e7758. doi: 10.7717/peerj.7758 (PMC6791347; doi:10.7717/peerj.7758)

Supplementary Material 2: SRA Validation of DRD_5 functionality in other mammals presenting low quality protein tag (LQ)

Ovis aries SRA confirmation of the absence of 1-nucleotide deletion

SRA experiments searched:
SRX2802995 - PRJNA386429 - Inner Mongolia Agricultural University
SRX2802991 - PRJNA386429 - Inner Mongolia Agricultural University
SRX3903525 - PRJNA433439 - Gansu Agricultural University

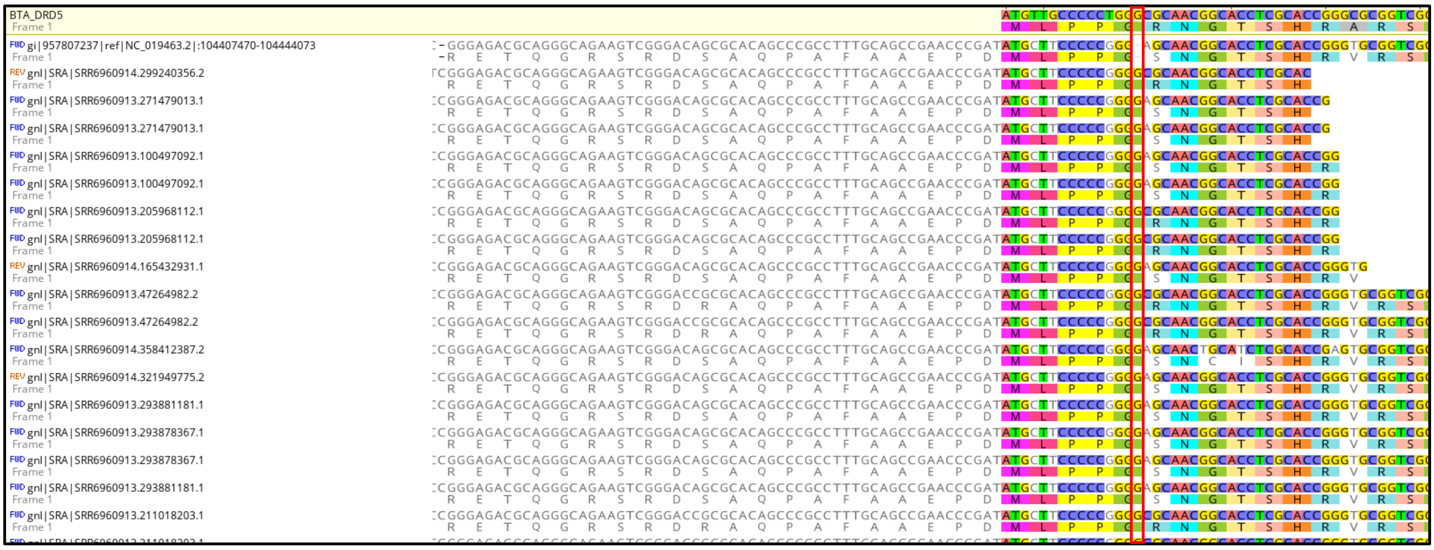

Ovis aries SRA confirmation of the absence of 2-nucleotide deletion

SRA experiments searched:
SRX2802995 - PRJNA386429 - Inner Mongolia Agricultural University
SRX2802991 - PRJNA386429 - Inner Mongolia Agricultural University
SRX3903525 - PRJNA433439 - Gansu Agricultural University

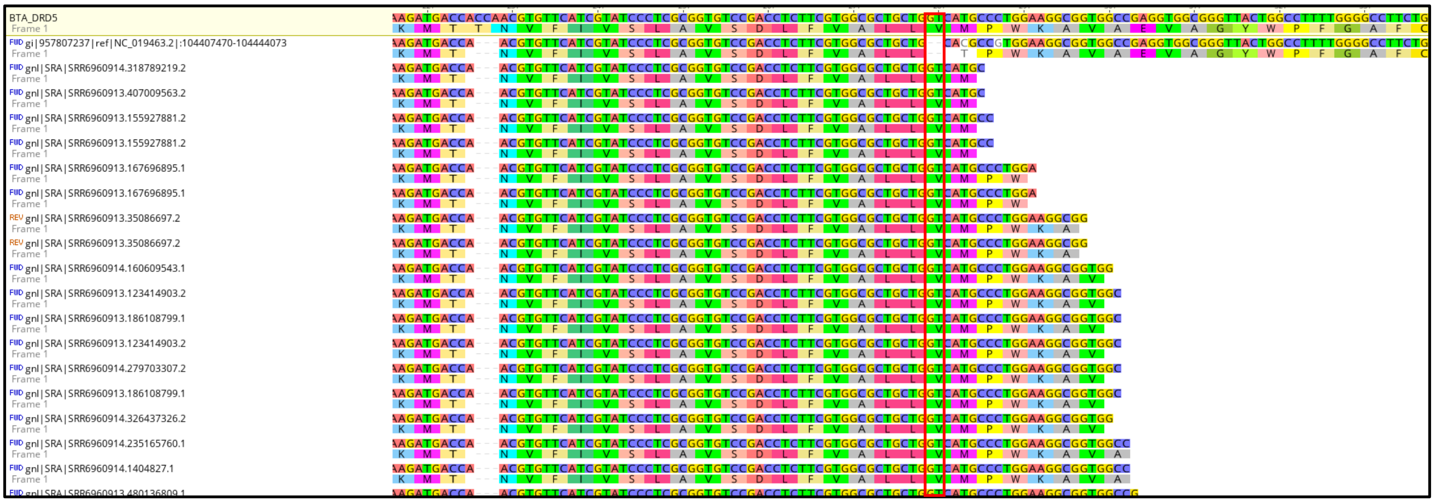

Bison bison bison SRA confirmation of the absence of 1-nucleotide insertion

SRA experiment searched:
SRX1766681 - PRJNA321590 - Sichuan university

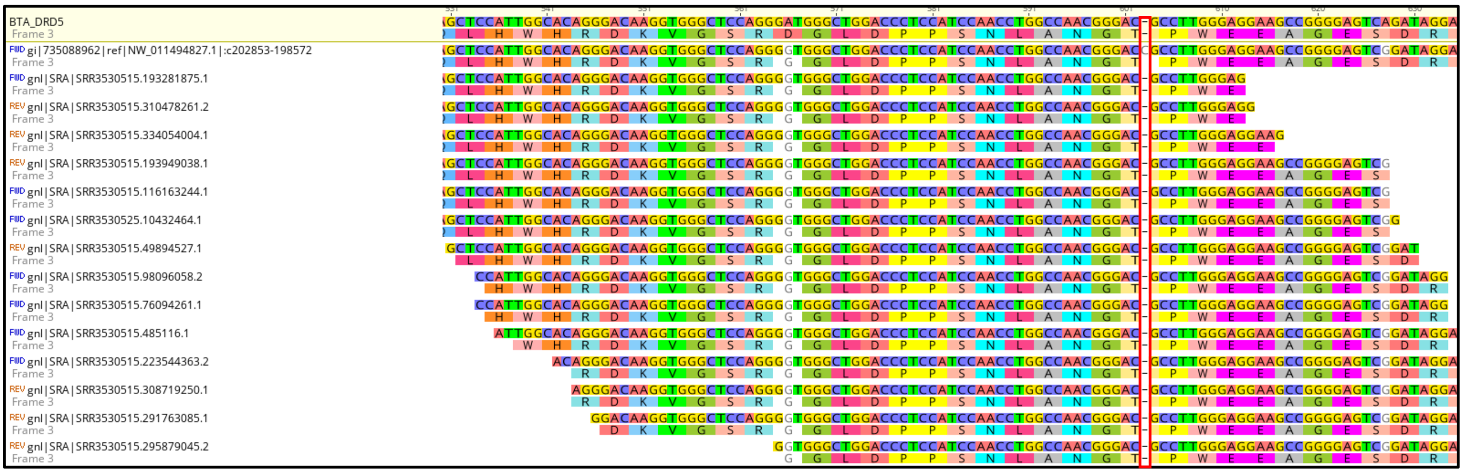

Phascolarctos cinereus SRA confirmation of the absence of 1 nucleotide deletion

SRA experiments searched:
ERX376153 - PRJEB5196 – BRAEMBL
ERX1942092 - PRJEB19982 - Koala genome consortium

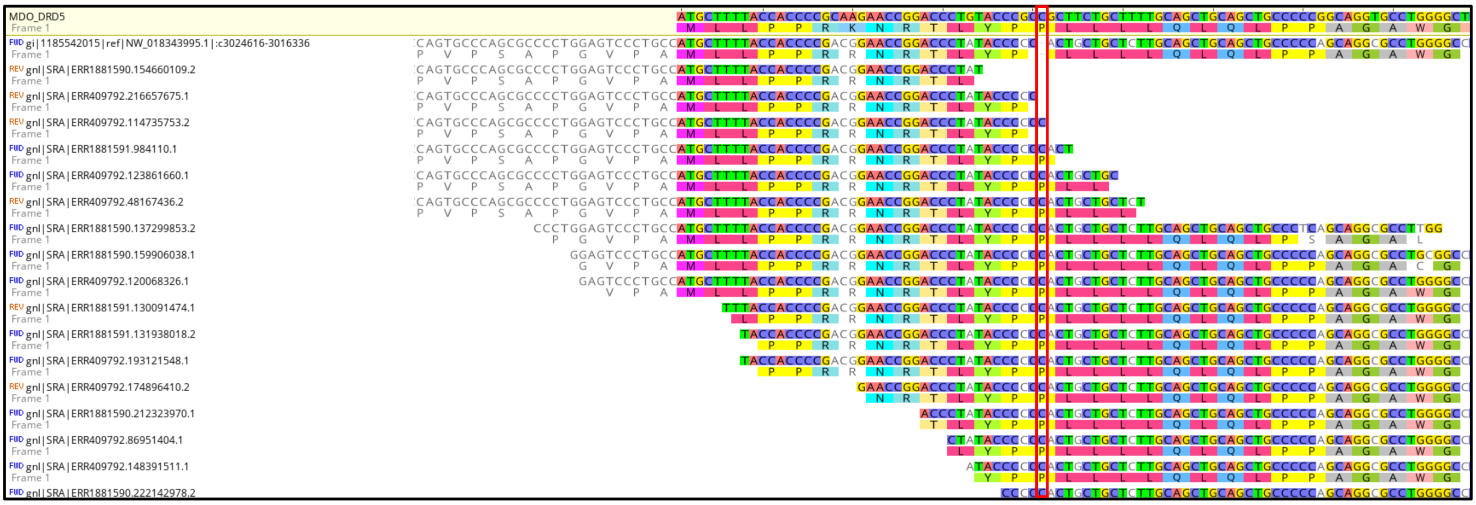

Ochotona princeps SRA reads supporting a premature stop codon and reads supporting its absence in the same species

SRA experiment searched:
Entire set of SRX experiments from the BioProject PRJNA74593 - Broad Institute

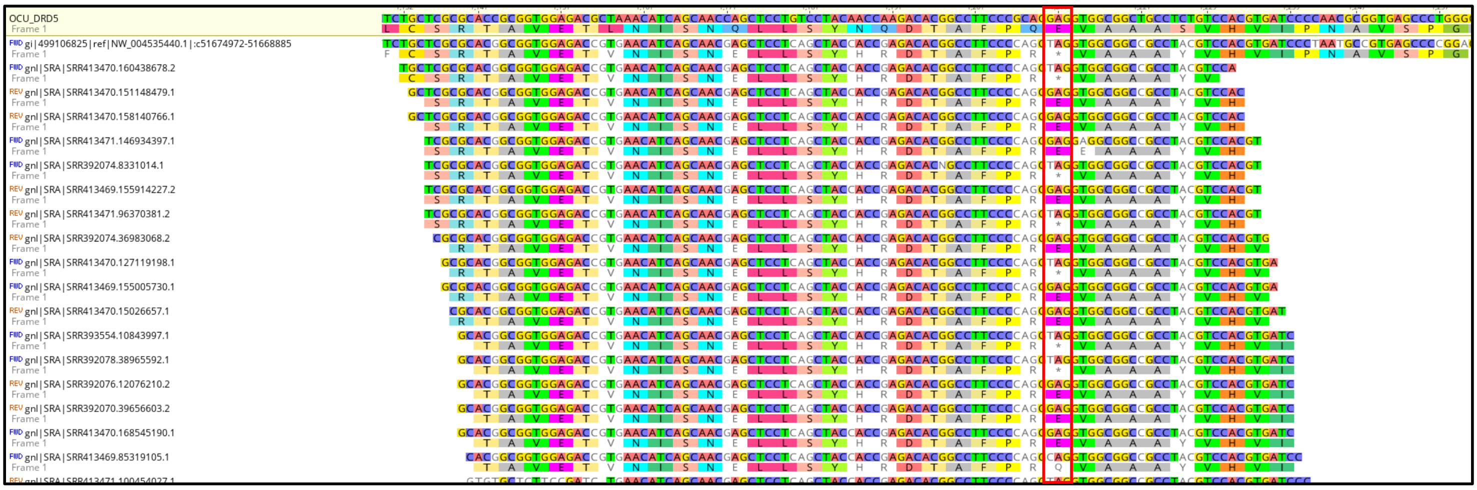

Supplement: Supplemental Information 4 — - [file peerj-07-7758-s004.pdf]
