# Supplementary material for: The dopamine receptor D5 gene shows signs of independent erosion in toothed and baleen whales
Source: PeerJ. 2019 Oct 11;7:e7758. doi: 10.7717/peerj.7758 (PMC6791347; doi:10.7717/peerj.7758)

Supplementary Material 3: SRA Validation of 1-nucleotide insertion in DRD_5 of *Chrysochloris asiatica*

SRA experiments searched:

SRX110136 - PRJNA74591 - Broad Institute (BI)

SRX110137 - PRJNA74591 - Broad Institute (BI)

SRX110138 - PRJNA74591 - Broad Institute (BI)

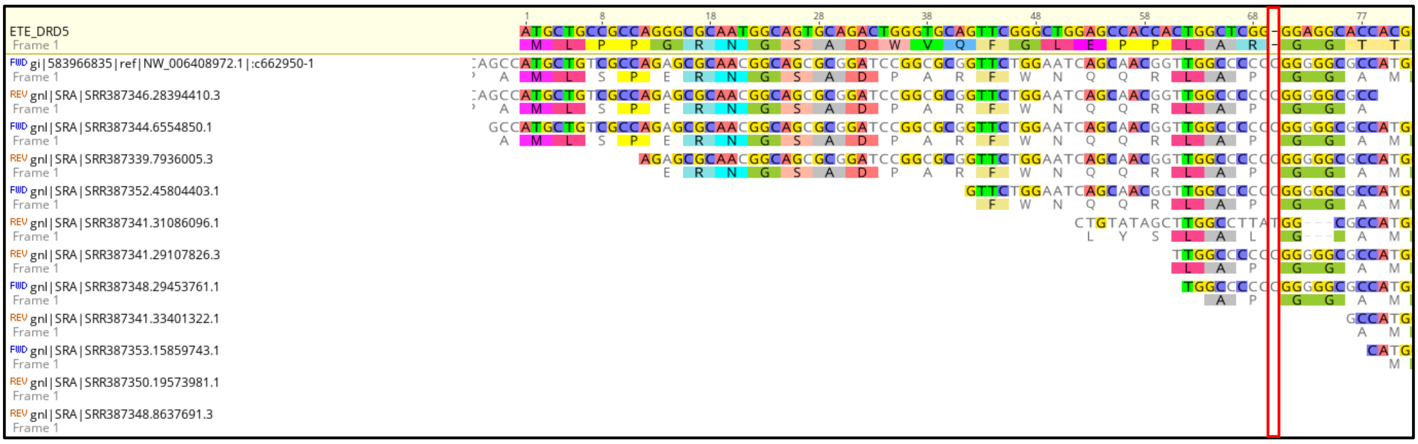

Supplement: Supplemental Information 5 — - [file peerj-07-7758-s005.pdf]
