# Supplementary material for: The dopamine receptor D5 gene shows signs of independent erosion in toothed and baleen whales
Source: PeerJ. 2019 Oct 11;7:e7758. doi: 10.7717/peerj.7758 (PMC6791347; doi:10.7717/peerj.7758)

Supplementary Material 4: Maximum Likelihood (ML) phylogram concerning DRD_5 sequences from Mysticeti and Odontoceti species, as well as from *Hippopotamus amphibius* (hippopotamus), *Bos taurus* (cattle) and *Homo sapiens* (human). Adjacent numbers to nodes depict the corresponding bootstrap support value (BS) - 1000. *Homo sapiens* (human) is set as outgroup.

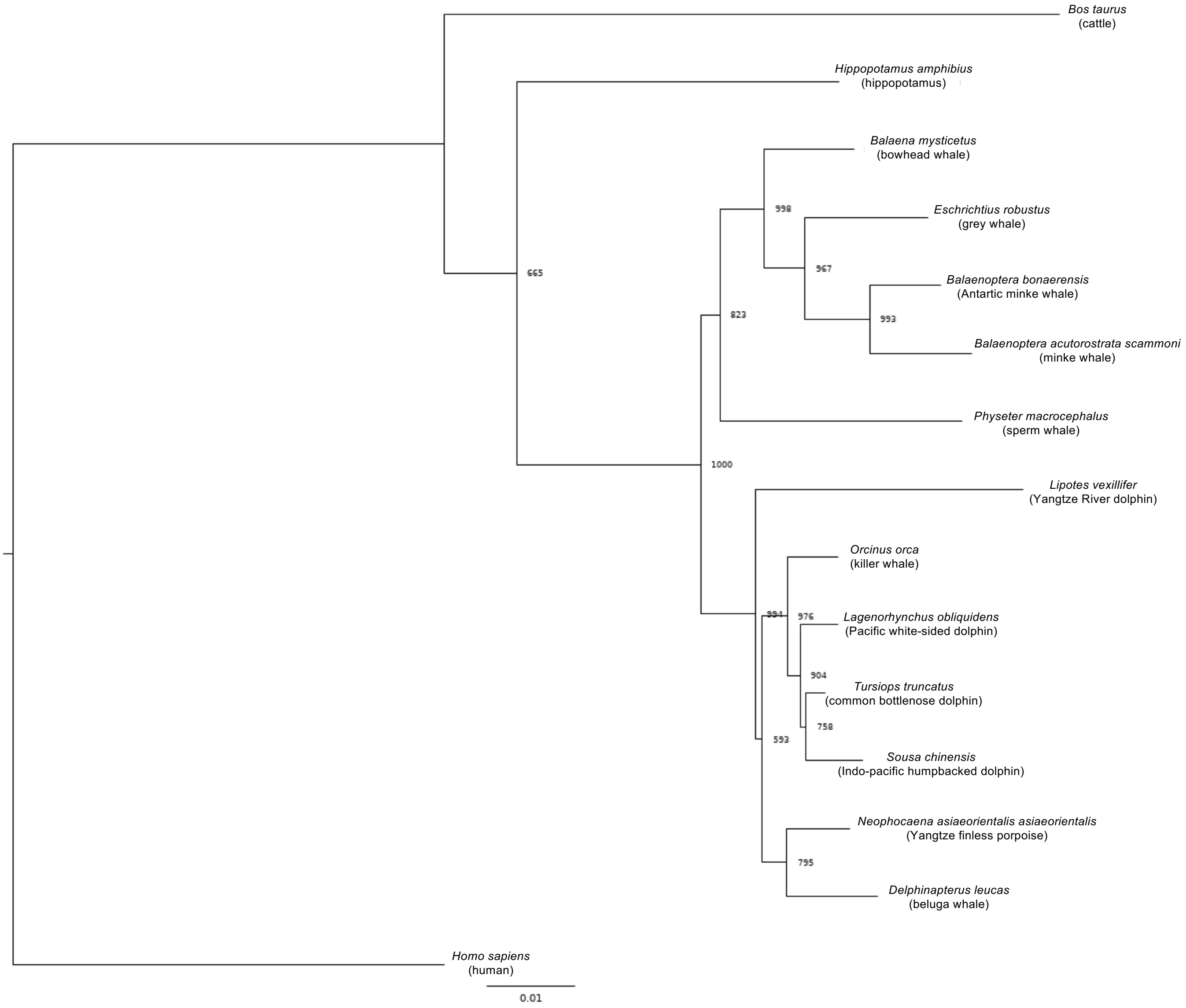

Supplement: Supplemental Information 6 — Adjacent numbers to nodes depict the corresponding bootstrap support value (BS). Homo sapiens (human) is set as outgroup. [file peerj-07-7758-s006.pdf]
