# Supplementary material for: The dopamine receptor D5 gene shows signs of independent erosion in toothed and baleen whales
Source: PeerJ. 2019 Oct 11;7:e7758. doi: 10.7717/peerj.7758 (PMC6791347; doi:10.7717/peerj.7758)
Supplement: Supplemental Information 8 — - [file peerj-07-7758-s008.docx]

| **Supplementary Table 1:** Selected reference species used in the sequence retrieval and/or gene annotation procedures of non-cetacean mammals with the respective DRD_5_ accession numbers. | | | | |  |
| --- | --- | --- | --- | --- | --- |
| **#** |  | **Test Species** | **Reference Species** | **Reference Species DRD_5_** **Accession number** |  |
| 1 |  | [*Microcebus murinus*](https://www.ncbi.nlm.nih.gov/Taxonomy/Browser/wwwtax.cgi?mode=Info&id=30608&lvl=3&lin=f&keep=1&srchmode=1&unlock) | Otolemur garnettii | XM_003800208.3 |  |
| 2 |  | *Jaculus jaculus* | *Mus musculus* | NM_013503.3 |  |
| 3 |  | *Chrysochloris asiatica* | *Echinops telfairi* | XM_004715080.1 |  |
| 4 |  | *Elephantulus edwardii* | *Orycteropus afer afer* | XM_007948779.1 |  |
| 5 |  | *Erinaceus europaeus* | *Sorex araneus* | XM_004617490.1 |  |
| 6 |  | *Condylura cristata* | *Sorex araneus* | XM_004617490.1 |  |
| 7 |  | *Ovis aries* | *Bos taurus* | NM_001206629.3 |  |
| 8 |  | *Bison bison bison* | *Bos taurus* | NM_001206629.3 |  |
| 9 |  | *Phascolarctos cinereus* | *Monodelphis domestica* | XM_001371240.2 |  |
| 10 |  | *Ochotona princeps* | *Oryctolagus cuniculus* | XM_008274145.2 |  |
| 11 |  | *Myotis davidii* | *Desmodus rotundus* | XM_024573892.1 |  |
