# Supplementary material for: The dopamine receptor D5 gene shows signs of independent erosion in toothed and baleen whales
Source: PeerJ. 2019 Oct 11;7:e7758. doi: 10.7717/peerj.7758 (PMC6791347; doi:10.7717/peerj.7758)
Supplement: Supplemental Information 9 — - [file peerj-07-7758-s009.docx]

| **Supplementary Table 2:** Accession numbers of the mammalian analysed sequences * tagged low-quality, ^a^ genomes without annotation, (ᴪ) pseudogenised. | | | | |  |
| --- | --- | --- | --- | --- | --- |
| **#** |  | **Species** | **Order** | **Accession number** |  |
| 1 | HSA | *Homo sapiens* | Primate-Hominoidae | NM_000798.4 |  |
| 2 | GGO | *Gorilla gorilla gorilla* | Primate-Hominoidae | XM_004038436.2 |  |
| 3 | NLE | *Nomascus leucogenys* | Primate-Hominoidae | XM_012499761.1 |  |
| 4 | PPA | *Pan paniscus* | Primate-Hominoidae | XM_003826782.2 |  |
| 5 | PTR | *Pan troglodytes* | Primate-Hominoidae | XM_528711.6 |  |
| 6 | PAB | *Pongo abelii* | Primate-Hominoidae | XM_002814584.3 |  |
| 7 | MLE | *Mandrillus leucophaeus* | Primate-Cercopithecoidea | XM_011987817.1 |  |
| 8 | MNE | *Macaca nemestrina* | Primate-Cercopithecoidea | XM_011714502.2 |  |
| 9 | MMU | *Macaca mulatta* | Primate-Cercopithecoidea | XM_001097117.3 |  |
| 10 | MFA | *Macaca fascicularis* | Primate-Cercopithecoidea | XM_005554477.2 |  |
| 11 | RRO | *Rhinopithecus roxellana* | Primate-Cercopithecoidea | XM_010357591.1 |  |
| 12 | RBI | *Rhinopithecus bieti* | Primate-Cercopithecoidea | XM_017871844.1 |  |
| 13 | CAT | *Cercocebus atys* | Primate-Cercopithecoidea | XM_012045471.1 |  |
| 14 | CSA | *Chlorocebus sabaeus* | Primate-Cercopithecoidea | XM_008017903.1 |  |
| 15 | CAN | *Colobus angolensis palliatus* | Primate-Cercopithecoidea | XM_011935438.1 |  |
| 16 | PAN | *Papio anubis* | Primate-Cercopithecoidea | XM_003898477.2 |  |
| 17 | PTE | *Piliocolobus tephrosceles* | Primate-Cercopithecoidea | XM_023206702.2 |  |
| 18 | THG | *Theropithecus gelada* | Primate-Cercopithecoidea | XM_025385369.1 |  |
| 19 | ANA | *Aotus nancymaae* | Primate-Platyrrhini | XM_012466993.2 |  |
| 20 | SBO | *Saimiri boliviensis boliviensis* | Primate-Platyrrhini | XM_003934678.2 |  |
| 21 | CCA | *Cebus capucinus imitator* | Primate-Platyrrhini | XM_017539167.1 |  |
| 22 | CJA | *Callithrix jacchus* | Primate-Platyrrhini | XM_002745992.3 |  |
| 23 | OGA | *Otolemur garnettii* | Primate-Strepsirrhini | XM_003800208.3 |  |
| 24 | MMU | *Microcebus murinus* | Primate-Strepsirrhini | Gene not annotated. Unknown coding status. |  |
| 25 | CSY | *Carlito syrichta* | Primate-Haplorrhini | XM_008061093.2 |  |
| 26 | TCH | *Tupaia chinensis* | Scandentia | XM_014581979.1 |  |
| 27 | GVA | *Galeopterus variegatus* | Dermoptera | XM_008571486.1 |  |
| 28 | NGA | *Nannospalax galili* | Rodentia-Myomorpha | XM_008833111.1 |  |
| 29 | CGR | *Cricetulus griseus* | Rodentia-Myomorpha | XM_003496643.2 |  |
| 30 | PMA | *Peromyscus maniculatus bairdii* | Rodentia-Myomorpha | XM_006985622.2 |  |
| 31 | MOC | *Microtus ochrogaster* | Rodentia-Myomorpha | XM_005365966.2 |  |
| 32 | MMU | *Mus musculus* | Rodentia-Myomorpha | NM_013503.3 |  |
| 33 | MPA | *Mus paharis* | Rodentia-Myomorpha | XM_021211215.1 |  |
| 34 | MCA | *Mus caroli* | Rodentia-Myomorpha | XM_021163858.1 |  |
| 35 | RNO | *Rattus norvegicus* | Rodentia-Myomorpha | NM_012768.1 |  |
| 36 | MAU | *Mesocricetus auratus* | Rodentia-Myomorpha | XM_005068824.3 |  |
| 37 | MEU | *Meriones unguiculatus* | Rodentia-Myomorpha | XM_021649643.1 |  |
| 38 | JJA | Jaculus jaculus | Rodentia-Myomorpha | Gene not annotated. Unknown coding status. |  |
| 39 | ITR | *Ictidomys tridecemlineatus* | Rodentia-Sciuromorpha | XM_005319077.2 |  |
| 40 | MMA | *Marmota marmota marmota* | Rodentia-Sciuromorpha | XM_015481961.1 |  |
| 41 | MFL | *Marmota flaviventris* | Rodentia-Sciuromorpha | XM_027939494.1 |  |
| 42 | UPA | *Urocitellus parryii* | Rodentia-Sciuromorpha | XM_026403213.1 |  |
| 43 | DOR | *Dipodomys ordii* | Rodentia-Castorimorpha | XM_013023905.1 |  |
| 44 | CCAN | *Castor canadensis* | Rodentia-Castorimorpha | XM_020178366.1 |  |
| 45 | CPO | *Cavia porcellus* | Rodentia-Hystricomorpha | XM_003464368.2 |  |
| 46 | HGL | *Heterocephalus glaber* | Rodentia-Hystricomorpha | XM_004847511.2 |  |
| 47 | ODE | *Octodon degus* | Rodentia-Hystricomorpha | XM_004624567.1 |  |
| 48 | FDA | *Fukomys damarensis* | Rodentia-Hystricomorpha | XM_010641931.1 |  |
| 49 | CLA | *Chinchilla lanigera* | Rodentia-Hystricomorpha | XM_005408842.1 |  |
| 50 | OCU | *Oryctolagus cuniculus* | Lagomorpha | XM_008274145.2 |  |
| 51 | OPR | *Ochotona princeps** | Lagomorpha | XM_012931415.1 |  |
| 52 | SSC | *Sus scrofa* | Cetartiodactyla -Suina | XM_013989284.2 |  |
| 53 | VPA | *Vicugna pacos* | Cetartiodactyla-Camelidae | XM_006216382.2 |  |
| 54 | CFE | *Camelus ferus* | Cetartiodactyla-Camelidae | XM_006189051.2 |  |
| 55 | CBA | *Camelus bactrianus* | Cetartiodactyla-Camelidae | XM_010973032.1 |  |
| 56 | CDR | *Camelus dromedarius* | Cetartiodactyla-Camelidae | XM_010975357.1 |  |
| 57 | BMU | *Bos mutus* | Cetartiodactyla-Bovinae | XM_005893192.1 |  |
| 58 | BBI | *Bison bison bison** | Cetartiodactyla-Bovinae | XM_010847779.1 |  |
| 59 | BTA | *Bos taurus* | Cetartiodactyla-Bovinae | NM_001206629.3 |  |
| 60 | BBU | *Bubalus bubalis* | Cetartiodactyla-Bovinae | XM_006043819.2 |  |
| 61 | OAR | *Ovis aries** | Cetartiodactyla-Caprinae | XM_015096298.1 |  |
| 62 | CHI | *Capra hircus* | Cetartiodactyla-Caprinae | XM_005681898.2 |  |
| 63 | OVI | *Odocoileus virginianus texanus* | Cetartiodactyla-Cervidae | XM_020908699.1 |  |
| 64 | PHO | *Pantholops hodgsonii* | Cetartiodactyla-Antilopinae | XM_005962148.1 |  |
| 65 | OOR | *Orcinus orca** | Cetartiodactyla-Cetacea | XM_004286766.2 (ᴪ) |  |
| 66 | TTR | *Tursiops truncatus* | Cetartiodactyla-Cetacea | XM_019928014.1  QUXD02011962.1 – Genomic Contig used in the manual prediction. (ᴪ) |  |
| 67 | LVE | *Lipotes vexillifer* | Cetartiodactyla-Cetacea | Gene not annotated, manual prediction. (ᴪ) |  |
| 68 | SCH^a^ | *Sousa chinensis* | Cetartiodactyla-Cetacea | QWLN01021157.1 (ᴪ) |  |
| 69 | LOB | *Lagenorhynchus obliquidens** | Cetartiodactyla-Cetacea | XM_027102813.1 (ᴪ) |  |
| 70 | DLE | *Delphinapterus leucas** | Cetartiodactyla-Cetacea | XM_022567155.1 (ᴪ) |  |
| 71 | NAS | *Neophocaena asiaeorientalis asiaeorientalis** | Cetartiodactyla-Cetacea | XM_024741783.1 (ᴪ) |  |
| 72 | PMA | *Physeter macrocephalus** | Cetartiodactyla-Cetacea | XM_024119537.1 (ᴪ) |  |
| 73 | BAC | *Balaenoptera acutorostrata scammoni* | Cetartiodactyla-Cetacea | Gene not annotated, manual prediction. Unknown coding status. |  |
| 74 | BBO^a^ | *Balaenoptera bonaerensis* | Cetartiodactyla-Cetacea | DF554641.1 (ᴪ) |  |
| 75 | ERO^a^ | *Eschrichtius robustus* | Cetartiodactyla-Cetacea | NIPP01013563.1 (ᴪ) |  |
| 76 | BMY^a^ | *Balaena mysticetus* | Cetartiodactyla-Cetacea | gnl\|BL_ORD_ID\|3417 (ᴪ) (downloaded from bowhead-whale.org) |  |
| 77 | HAM^a^ | *Hippopotamus amphibius* | Cetartiodactyla-Hippopotamidae | NKPW01000739.1 |  |
| 78 | PCI | *Phascolarctos cinereus** | Diprotodontia-[Phascolarctidae](https://www.ncbi.nlm.nih.gov/Taxonomy/Browser/wwwtax.cgi?mode=Undef&id=38624&lvl=3&keep=1&srchmode=1&unlock) | XM_020992084.1 |  |
| 79 | VOU | *Vombatus ursinus* | Diprotodontia-Vombatidae | XM_027836427.1 |  |
| 80 | CSI | *Ceratotherium simum simum* | Perissodactyla-Rhinoceratidae | XM_004432348.2 |  |
| 81 | ECA | *Equus caballus* | Perissodactyla-Equidea | XM_005614781.3 |  |
| 82 | EPR | *Equus przewalskii* | Perissodactyla-Equidea | XM_008543661.1 |  |
| 83 | EAS | *Equus asinus* | Perissodactyla-Equidea | XM_014864319.1 |  |
| 84 | NSC | *Neomonachus*  *schauinslandi* | Carnivora-Caniformia | XM_021677594.1 |  |
| 85 | ORO | *Odobenus rosmarus divergens* | Carnivora-Caniformia | XM_004415889.2 |  |
| 86 | LWE | *Leptonychotes weddellii* | Carnivora-Caniformia | XM_006747176.1 |  |
| 87 | AME | *Ailuropoda melanoleuca* | Carnivora-Caniformia | XM_011229965.2 |  |
| 88 | UMA | *Ursus maritimus* | Carnivora-Caniformia | XM_008685829.1 |  |
| 89 | UAR | *Ursus arctos horribilis* | Carnivora-Caniformia | XM_026514401.1 |  |
| 90 | MFU | *Mustela putorius furo* | Carnivora-Caniformia | XM_004770551.1 |  |
| 91 | CFA | *Canis lupus familiaris* | Carnivora-Caniformia | XM_005618578.1 |  |
| 92 | CLU | *Canis lupus dingo* | Carnivora-Caniformia | XM_025438279.1 |  |
| 93 | VUV | *Vulpes vulpes* | Carnivora-Caniformia | XM_026002149.1 |  |
| 94 | CAU | *Callorhinus ursinus* | Carnivora-Caniformia | XM_025887601.1 |  |
| 95 | ELU | *Enhydra lutris kenyoni* | Carnivora-Caniformia | XM_022506347.1 |  |
| 96 | PALT | *Panthera tigris altaica* | Carnivora- Feliformia | XM_015534698.1 |  |
| 97 | FCA | *Felis catus* | Carnivora- Feliformia | XM_003985519.5 |  |
| 98 | AJU | *Acinonyx jubatus* | Carnivora- Feliformia | XM_015072964.2 |  |
| 99 | PPAR | *Panthera pardus* | Carnivora- Feliformia | XM_019453704.1 |  |
| 100 | PCO | *Puma concolor* | Carnivora- Feliformia | XM_025922066.1 |  |
| 101 | ZCA | *Zalophus californianus* | Carnivora-[Otariidae](https://www.ncbi.nlm.nih.gov/Taxonomy/Browser/wwwtax.cgi?mode=Undef&id=9702&lvl=3&keep=1&srchmode=1&unlock) | XM_027599538.1 |  |
| 102 | PVA | *Pteropus vampyrus* | Chiroptera | XM_011374674.1 |  |
| 103 | EFU | *Eptesicus fuscus* | Chiroptera | XM_008150879.1 |  |
| 104 | PALE | *Pteropus alecto* | Chiroptera | XM_015594847.1 |  |
| 105 | MDA | *Myotis davidii** | Chiroptera | XM_006779351.2 |  |
| 106 | MBR | *Myotis brandtii* | Chiroptera | XM_005866153.2 |  |
| 107 | MNA | *Miniopterus natalensis* | Chiroptera | XM_016203704.1 |  |
| 108 | MLU | *Myotis lucifugus* | Chiroptera | XM_006106071.3 |  |
| 109 | DRO | *Desmodus rotundus* | Chiroptera | XM_024573892.1 |  |
| 110 | RSI | *Rhinolophus sinicus* | Chiroptera | XM_019716569.1 |  |
| 111 | HAR | *Hipposideros armiger* | Chiroptera | XM_019661154.1 |  |
| 112 | LAF | *Loxodonta africana* | Afrotheria-Proboscidea | XM_003411269.3 |  |
| 113 | TMA | *Trichechus manatus latirostris* | Afrotheria-Sirenia | XM_004385912.3 |  |
| 114 | ETE | *Echinops telfairi* | Afrotheria-Tenrecidae | XM_004715080.1 |  |
| 115 | OAF | *Orycteropus afer afer* | Afrotheria-Tubulidentata | XM_007948779.1 |  |
| 116 | CAS | *Chrysochloris asiatica* | Afrotheria-Chrysochloridae | Gene not annotated, manual prediction. (ᴪ) |  |
| 117 | EED | *Elephantulus edwardii* | Afrotheria-Macroscelidea | Gene not annotated, manual prediction. |  |
| 118 | MJA | *Manis javanica* | Pholidota | XM_017640904.1 |  |
| 119 | SAR | *Sorex araneus* | Eulipotyphla | XM_004617490.1 |  |
| 120 | EEU | *Erinaceus europaeus* | Eulipotyphla | Gene not annotated. Unknown coding status. |  |
| 121 | CCR | Condylura cristata | Eulipotyphla | Gene not annotated. Unknown coding status. |  |
| 122 | DNO | *Dasypus novemcinctus* | Cingulata | XM_004468905.2 |  |
| 123 | SHA | *Sarcophilus harrisii* | Dasyuromorphia | XM_012552332.2 |  |
| 124 | MDO | *Monodelphis domestica* | Didelphidae | XM_001371240.2 |  |
